# Life history alterations upon oral and hemocoelic bacterial exposure in the butterfly *Melitaea cinxia*

**DOI:** 10.1002/ece3.5586

**Published:** 2019-08-28

**Authors:** Luisa Woestmann, Dimitri Stucki, Marjo Saastamoinen

**Affiliations:** ^1^ Organismal and Evolutionary Biology Research Programme Faculty of Biological and Environmental Sciences University of Helsinki Helsinki Finland

**Keywords:** fecundity, gene expression, Glanville fritillary butterfly, immune response, wounding

## Abstract

Life history strategies often shape biological interactions by specifying the parameters for possible encounters, such as the timing, frequency, or way of exposure to parasites. Consequentially, alterations in life‐history strategies are closely intertwined with such interaction processes. Understanding the connection between life‐history alterations and host–parasite interactions can therefore be important to unveil potential links between adaptation to environmental change and changes in interaction processes. Here, we studied how two different host–parasite interaction processes, oral and hemocoelic exposure to bacteria, affect various life histories of the Glanville fritillary butterfly *Melitaea cinxia*. We either fed or injected adult butterflies with the bacterium *Micrococcus luteus* and observed for differences in immune defenses, reproductive life histories, and longevity, compared to control exposures. Our results indicate differences in how female butterflies adapt to the two exposure types. Orally infected females showed a reduction in clutch size and an earlier onset of reproduction, whereas a reduction in egg weight was observed for hemocoelically exposed females. Both exposure types also led to shorter intervals between clutches and a reduced life span. These results indicate a relationship between host–parasite interactions and changes in life‐history strategies. This relationship could cast restrictions on the ability to adapt to new environments and consequentially influence the population dynamics of a species in changing environmental conditions.

## Graphical abstract

Here, we report differences in how female butterflies adapt life‐history traits upon two distinct infection modes. Oral exposure resulted in a accelerated decrease of clutch sizes but an increase in clutch number, whereas hemocoelic exposure resulted in a reduction of egg weight.

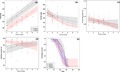

## 1 INTRODUCTION

The world is changing. Consequently, also the conditions for biological interactions (conflict and cooperation) may change. Biological interactions are often key drivers in population dynamics, for example, through predator–prey dynamics (Gilg, Sittler, & Hanski, 2009), sexual conflicts (Rankin & Kokko, 2007), or parasite pressure (Hatcher, Dick, & Dunn, 2006). A major component of population dynamics are life histories (Jaspers, Marty, & Kiørboe, 2018; Sæther et al., 2013): traits that determine how many individuals enter a population via reproduction or dispersal, how fast they reach reproductive age, and how likely they are to survive to the different life stages. Given the interplay between the life histories and population dynamics (Stearns, 1976), maintaining optimal fitness in changing environmental conditions may require shifts in either or both of these traits (Dunham & Overall, 1994; van de Pol et al., 2010; Williams, Jacquemyn, Ochocki, Brys, & Miller, 2015).

One of the most important biological interactions that drives individual life histories is the conflict between hosts and parasites. Although life histories usually adapt on an evolutionary scale (Flat & Heyland, 2011), parasitic infections can also induce short‐term changes in life‐history traits through plasticity. For example, host–parasite interactions have been shown to reduce general life span (e.g., Dainat, Evans, Chen, Gauthier, & Neumann, 2012; Polak & Starmer, 1998) and both male and female fecundity (e.g., Bollache, Rigaud, & Cézilly, 2011; Worden, Parker, & Pappas, 2000). Although plasticity may allow an individual to adapt to many conditions, plasticity itself is not endless and often comes with trade‐offs (DeWitt, Sih, & Wilson, 1998; Murren et al., 2015). As a consequence, the ability to adapt to changes in the environment may depend on how a species (or population) resolves the conflict with parasites and how this resolution affects life‐history traits.

Part of this resolution constitutes the hosts' immune defense. Most organisms are able to mount an immune response against parasites, which often is energetically costly or incurs other costs in the form of life‐history trade‐offs (Flat & Heyland, 2011; Schmid‐Hempel, 2011). Bacterial pathogens can be opposed by expression of effector molecules such as antimicrobial peptides. For example, the Toll and Imd pathways are specifically effective against Gram‐positive and Gram‐negative bacteria, respectively (De Gregorio, Spellman, Tzou, Rubin, & Lemaitre, 2002). Each of the two pathways responds to molecular patterns associated with the specific type of bacteria and ultimately leads to the expression of antimicrobial peptides that destroy or help to destroy the respective bacteria. The immune response against multicellular parasites or, sometimes, aggregations of bacteria, constitutes encapsulation, which often is connected to the prophenoloxidase immune pathway (Cerenius & Söderhäll, 2004; Strand, 2008). The encapsulation response results in the enclosure of parasites in a dense capsule consisting of hemocytes and ultimately leads to suffocation of the parasite or destruction through oxygen radicals. Often, the capsule is further coated in layers of melanin (Strand, 2008). This melanization process is also involved in wound healing in insects (Theopold, Li, Fabbri, Scherfer, & Schmidt, 2002). Localization as well as the extent of the immune response is usually determined by expression of signaling molecules of the specific immune pathway at the site of infection (Ferrandon, Imler, Hetru, & Hoffmann, 2007).

For most bacterial infections in insects, the site of entry is either the gut, if the bacteria were ingested via the food, or the hemocoel, if the bacteria entered through a wound in the cuticle (Vallet‐Gely, Lemaitre, & Boccard, 2008). As the pathogenicity of bacteria may depend on the mode of infection, the extent of the immune response may depend on whether the host is exposed to bacteria orally or through the hemocoel (e.g., Banerjee & Dangar, 1995). Experimental selection in *Drosophila melanogaster* has shown fast evolutionary adaptation of the host's immune system to a specific route of infection by bacteria (Faria et al., 2015; Martins, Faria, Teixeira, Magalhães, & Sucena, 2013). Similarly, genetic variation in the immune response to oral and hemocoelic infection can vary among populations (Behrens et al., 2014). As immune defenses and infections often come with trade‐offs (Flat & Heyland, 2011; Schmid‐Hempel, 2011), we consider it likely that the infection route could also affect how individuals express life histories. Phenotypic plasticity has already been shown for horizontal versus vertical transmission in *Daphnia magna* (Vizoso & Ebert, 2005), whereas research on oral versus hemocoelic transmission is currently scarce.

Here, we investigated the impact of infection by bacteria on the immune response and on a set of life history traits in the butterfly *Melitaea cinxia*. We exposed female butterflies to the bacterial pathogen *Micrococcus luteus*, which is commonly used in studies on entomopathology (e.g., Ahmed, Baggott, Maingon, & Hurd, 2002; Freitak et al., 2014; Kajla, Andreeva, Gilbreath, & Paskewitz, 2010) and was established previously for this model system (Woestmann, Kvist, & Saastamoinen, 2016). Exposure to the pathogen occurred either through feeding (oral exposure) or through injection (hemocoelic exposure). We assessed the impact of exposure on the total life span of the butterflies, as well as on the fecundity of the females. Furthermore, in order to get an understanding of the dynamics of the immune response, we measured the extent of the encapsulation response and the expression of genes reflecting the different immune pathways at two different time points after exposure. As *M. luteus* are Gram‐positive bacteria, we measured gene expression levels of two genes that are downstream of the Toll pathway: *lysozyme C*, which is regulated by the Toll pathway but responds to both Gram‐positive and Gram‐negative bacteria (Anderson & Cook, 1979; Dunn, 1986), and *pelle*, a signaling molecule that regulates the expression of antimicrobial peptides (Daigneault, Klemetsaune, & Wasserman, 2013). To investigate whether bacterial infection also affects the defense against other pathogens, we additionally measured expression of two genes downstream of the Imd pathway: *peptidoglycan recognition protein LC* (*PGRP‐LC*), which binds to peptidoglycans of the bacterial cell wall (Tanaka et al., 2008; Zaidman‐Rémy et al., 2011), and the antimicrobial effector peptide *attacin* (Carlsson, Nyström, Cock, & Bennich, 1998). Similarly, to reflect the status of immune defenses against nonbacterial pathogens, we additionally included the gene *β‐1,3‐glucan recognition protein*, which binds to the fungal cell wall component β‐1,3‐glucan (Lemaitre & Hoffmann, 2007), and two genes of the melanization process: *prophenoloxidase* (*proPO*), a inactive precursor to phenoloxidase, an enzyme responsible for the conversion of phenols to melanin (Söderhäll & Cerenius, 1998), and *serpin 3a* (Wang et al., 2017), a serine proteinase that acts as an inhibitor and regulator of the prophenoloxidase pathway.

## 2 MATERIAL AND METHODS

### 2.1 Study animals

Females of the Glanville fritillary butterfly (*M. cinxia*) carry the full number of oocytes in their ovarioles upon enclosure, which they lay in several clutches on the host plants (Boggs & Nieminen, 2004). A subset of the eggs are already mature at eclosion, while the rest mature during the adult stage (Wheat et al., 2011). Males and females have very different life histories: Most females mate just once, have a longer life span than males, and are more dispersive, whereas males often mate multiple times, engage in male–male competition over females, and tend to stay more in the natal patch (Boggs & Nieminen, 2004; Duplouy, Ikonen, & Hanski, 2013; Kuussaari, Nieminen, & Hanski, 1996). For the experiments, we used first‐generation offspring from individuals that were collected as larvae from the wild, and were reared and mated in the laboratory. All larvae were reared on ad libitum* Plantago lanceolata* in climate chambers (28:15°C, D:N). The individuals were kept in nest boxes in their original family groups.

### 2.2 Experimental design

The study was divided into two major parts to test the effects of (a) oral and (b) hemocoelic bacterial exposure on the immune response of adult *M. cinxia*, as well as the effect of each exposure type on life histories.

#### 2.2.1 Oral bacterial exposure (OE)

##### Immune response setup

Adult female butterflies from 30 families (*n* = 477) were randomly divided into three oral exposure treatments (control, 5, and 10 mg/ml). All individuals were reared ad libitum on a 20% honey:water solution (Golden Nectar; monikukkaishunaja; SeaGood Oy Fort Deli), which was refreshed daily. For the control exposure, the diet was left unchanged, whereas for each of the two bacteria exposures, the diet was supplemented with *M. luteus* (lyophilized bacteria; ATCC No. 4698; Sigma‐Aldrich) to a bacterial concentration of either 5 mg/ml or 10 mg/ml, respectively. The lower concentration (5 mg/ml) was selected as a comparison to the already established concentration for hemocoelic exposure (Woestmann et al., 2016). To test whether a higher concentration would lead to a more pronounced immune response, as bacteria first have to overcome the ineligible milieu of the gut (Vallet‐Gely et al., 2008), the amount was doubled for the higher concentration (10 mg/ml). The respective food was provided from the day after eclosion until death of the female. Only during mating, all individuals received the control food to avoid infection of the control males.

##### Gene expression

To assess gene expression of the selected immune‐related genes, a set of individuals (~11/sex/exposure) were killed at each of two time points, after 24 or 72 hr after the oral treatment was initiated. The earlier time point was chosen based on our knowledge that 20 hr after hemocoelic exposure, immune response genes have shown differences in expression levels (Woestmann et al., 2016). We included the later time point to test for differences in expression levels over time. Some AMPs, for example, show an acute‐phase profile while others a late and sustained expression pattern (Erler, Popp, & Lattorff, 2011; Johnston, Makarova, & Rolff, 2014; Lemaitre & Hoffmann, 2007), and lysozyme, for example, has been shown to be up‐regulated even up to 120 hr prior infection (Kajla et al., 2010). The sampled individuals were flash‐frozen in liquid nitrogen and stored in −80°C until RNA extraction from the thorax. RNA extraction and qPCR were performed as described by Woestmann et al. (2016) with the same immune and reference genes.

##### Encapsulation response

In order to measure encapsulation response, we used a nylon microfilament to simulate infection by a parasitoid larva that is able to activate the encapsulation response of the immune system (Pölkki, Kortet, Hedrick, & Rantala, 2013). Encapsulation rate was measured 72 hr after the immune treatment had been initiated, on a separate set of individuals (~30/sex/exposure). The butterflies were immobilized for the measurement of the encapsulation response by spanning them on a soft sponge under a net. Through a hole in the net, the thorax is accessible. A small puncture was made in the middle of the thorax, and a 2 mm long piece of a nylon monofilament (diameter 0.18 mm), which was rubbed with sandpaper, was inserted into the butterfly and removed after 1 hr. A knot at the outer end of the filament guaranteed that the length of the inserted filament is equal for every individual. Measurement of the encapsulation response was performed as described by Saastamoinen and Rantala (2013). A subset of filaments was measured twice to test for repeatability (*n* = 24, *R* ^2^ = 0.995).

##### Female reproductive performance

To test for an effect of oral bacterial exposure on female reproductive performance, control or exposed females were mated to control males in a separate experiment. Adult female butterflies were fed ad libitum with a control 20% honey:water solution, which was either left unchanged for a control group (*n* = 23) or supplemented with *M. luteus* to a concentration of 5 mg/ml (*n* = 23). For logistic reasons, we only used the lower concentration for this experiment, as this was the already established concentration for this model system (Woestmann et al., 2016). Three days after the treatment, the females were mated to control males (*n* ~ 12) in a communal breeding cage (max. 20 individuals, Ø = 40cm, *h* = 50 cm) with randomly chosen individuals from the different families and avoidance of inbreeding. Nine control females and 10 bacteria‐exposed females (5 mg/ml) mated successfully. Males were on average three days old at the time of mating and were killed immediately thereafter. The mated females were placed individually into a small cage (13 × 20 cm) with a host plant (*P. lanceolata*) and fed ad libitum 20% honey:water, which was modified according to the allotted treatment (control/5 mg/ml). The host plants were checked daily for egg clutches until the females died. For every clutch, the number of eggs was counted and the entire clutch was weighted three days after laying. To assess hatching success for each clutch, the number of hatched larvae was counted on the day after the first larvae hatched.

##### Life span

To assess the effect of bacterial exposure on adult life span, we used an additional set of butterflies for oral exposure (*n* = 146 from 10 families) from a laboratory generation. The individuals were grouped into a control group (*n* = 48) and two bacterial exposure groups for the concentrations of 5 ml/mg (*n* = 49) and 10 ml/mg (*n* = 48), respectively. The individuals were kept as single groups (separated by treatment) in cylindrical cages with food ad libitum, and survival was checked daily.

#### 2.2.2 Hemocoelic bacterial exposure (HE)

##### Immune response setup

Adults (*n* = 324 from 11 families) were randomly divided among three hemocoelic exposure treatments (control, injection of PBS, and injection of bacteria). All individuals were fed ad libitum with a 20% honey:water solution, which was refreshed daily. Two days posteclosion, individuals from the PBS injection group were injected with 2 µl of 1xPBS solution, whereas individuals of the bacterial group got injected with 2 µl of *M. luteus* (5 mg/ml; concentration based on Woestmann et al., (2016)) dissolved in 1× PBS. Butterflies were immobilized for the injection by spanning them on a soft sponge under a net. Through a hole in the net, the thorax is accessible. Injection was performed with a Hamilton syringe (needle size 26s ga, bevel tip) by introducing the needle in a very low angle and only as deep as necessary to introduce the solution, to avoid extra damage of tissue. Control individuals experienced the same handling without the piercing to ensure similar stress levels.

##### Gene expression

As for the first experiment, a set of individuals (~11/sex/treatment) were killed at two time points (24 or 72 hr postinjection) to assess expression of the selected immune genes.

##### Encapsulation response

The encapsulation rate was also assessed as above for a set of individuals (~30/sex/treatment). The repeatability of the measurements was assessed by measuring a subset of filaments twice (*n* = 25, *R* ^2^ = 0.997).

##### Female reproductive performance

Adult female butterflies were allocated to either a control treatment (*n* = 19), PBS treatment (*n* = 19), or bacterial exposure treatment (*n* = 19). The treatments were applied as described above for the hemocoelic exposure. After 3 days, the females were mated to control males, which resulted in successful matings for 17 control females, 17 PBS‐injected females, and 18 bacteria‐exposed females, respectively.

##### Life span

To assess the effect of hemocoelic bacterial exposure on adult life span, we used an additional set of butterflies for hemocoelic exposure (*n* = 108 from 43 families) from a laboratory generation. The individuals were grouped into a control group (*n* = 55) and a bacterial exposure group (*n* = 54). We did not include a PBS injection group for the life span analyses to assess the effect of wounding itself due to the limited amount of individuals available for the experiment and as it has been previously shown to have no effect on longevity (Woestmann et al., 2016). The individuals were kept as single groups (separated by treatment) in cylindrical cages with food ad libitum, and survival was checked daily.

### 2.3 Statistical analysis

#### 2.3.1 Immunity

Gene expression was analyzed on normalized Ct scores. To normalize gene expression, we used the NORMA‐Gene algorithm, which does not require reference genes for normalization (Heckmann, Sørensen, Krogh, & Sørensen, 2011). As suggested by the authors, we nevertheless included the reference genes *Histone*, *mrpL7*, and* S28rpS24* to stabilize the normalization, but not as genes of interest. Hence, we do not report the results for these genes. For analysis, we inverted the Ct values because they are negatively correlated with specific transcript level (i.e., higher Ct values indicate lower gene expression levels).

We first assessed the option to use a principal component analysis, which was dissuaded by a low sampling adequacy for both oral (KMO: 0.46) and hemocoelic exposure (KMO: 0.46). As the removal of multiple genes to improve sampling adequacy would have resulted in multiple different analyses, we decided to directly perform a separate analysis for each gene. Separate models were run for each exposure type, oral exposure and hemocoelic exposure. All genes were first modeled in a linear mixed‐effects regression using the lme4 package (Bates, Maechler, Bolker, & Walker, 2014), with the inverted Ct value as response and treatment (for oral exposure: control, 5, 10 mg/ml; for hemocoelic exposure: control, PBS, 5 mg/ml) and time (24 and 72 hr postexposure) as fixed factors. Additionally, an interaction term between the two fixed factors was specified. Female family was specified as random effect. These models were only run to allow the subsequent pairwise analyses with the multcomp package (Hothorn, Bretz, & Westfall, 2008) and are not reported in the results. Because we were primarily interested in specific differences among the treatments, we performed a subset of multiple pairwise comparisons, irrespective of the significance of the interaction term. As we only analyzed a subset of all pairwise combinations, we did not adjust the resulting *p*‐values for multiple comparisons (Streiner, 2015); however, we did adjust all *p*‐values for the analysis of seven different genes, using false discovery rates (Benjamini & Hochberg, 1995). We only consider differences greater than one Ct (which corresponds to a twofold change in gene expression), irrespective of the associated statistical significance. We do so to avoid drawing far‐fetched conclusions outside of the explorative nature of this analysis.

Encapsulation response was analyzed in a linear mixed‐effects regression with the standardized gray value as response and treatment (oral exposure: control, 5, 10 mg/ml; hemocoelic exposure: control, PBS, 5 mg/ml). Female family was specified as random effect. As the comparison between the two concentrations was not of primary interest, we did not perform any post hoc analyses on the encapsulation response.

### 2.4 Life history

Egg laying date was analyzed in a linear mixed‐effects regression with day of the year as response, clutch number as fixed continuous covariate, and treatment (oral exposure: control, 5 mg/ml; hemocoelic exposure: control, PBS, 5 mg/ml) as fixed factor, and their interaction was included. Female id was specified as random effect to account for correlations among subsequent clutches of the same female. The number of clutches per female was analyzed in a generalized linear regression for oral exposure, using a generalized Poisson distribution from the VGAM package (Yee, 2018), due to strong under dispersion. In the case of hemocoelic exposure, generalized linear regression with a default Poisson distribution was used. For both, the amount of clutches per female was specified as response and treatment was specified as fixed factor. The number of eggs per clutch was analyzed in generalized linear mixed‐effects regression with a negative binomial distribution for both oral and hemocoelic expression. The amount of eggs per clutch was specified as response, clutch number as fixed continuous covariate, and treatment as fixed factor, and their interaction was included. Female id was specified as random effect. The weight of the eggs was analyzed in linear mixed‐effects regression, with the average weight per egg for each clutch as response, clutch number as fixed continuous covariate, treatment as fixed factor, an interaction term, and female id as random effect. Hatching success was analyzed in generalized linear mixed‐effects regression with a binomial distribution. Hatching (Yes/No) was specified as binary response variable, and we included clutch number as fixed continuous covariate, treatment as fixed factor, an interaction term between the two, and female id as random effect. Lastly, life span was analyzed in mixed‐effects Cox proportional hazard regression of the coxme package (Therneau, 2018), with day of death as response (no censoring occurred), female treatment as fixed factor, and female family as random effect. To assess the proportional hazard assumption, a normal Cox proportional hazard model of the survival package (Therneau, 2013) was specified with female family as frailty term, because this feature is not available from the coxme package. All other models were visually assessed for validity through inspection of the residuals and the leverage. Potential outliers were inspected by assessing their influence and the effect of their removal on the final outcome. If these effects were not too severe, we only removed the data points if our notes indicated an underlying reason for a bias. Based on these rules, no outliers were removed. In the life‐history traits after hemocoelic exposure, only PBS‐exposed females had more than four clutches. In order to improve the model fit, we removed these clutches for all models (four data points removed). Statistical analysis was carried out in R version 3.4.4 (R Core Team, 2018). In addition to those already referenced, we used the packages psych (Revelle, 2016), Hmisc (Harrell, 2018), lmerTest (Kuznetsova, Brockhoff, Bojesen, & Christensen, 2014), dplyr (Wickham, François, Henry, & Müller, 2019), tidyr (Wickham & Henry, 2018), lubridate (Grolemund & Wickham, 2011), stringr (Wickham, 2018), influence.ME (Nieuwenhuis, Te Grotenhuis, & Pelzer, 2012), and emmeans (Lenth, 2019).

## 3 RESULTS

### 3.1 Oral bacterial exposure

#### 3.1.1 Immune defense

**Figure 1:**
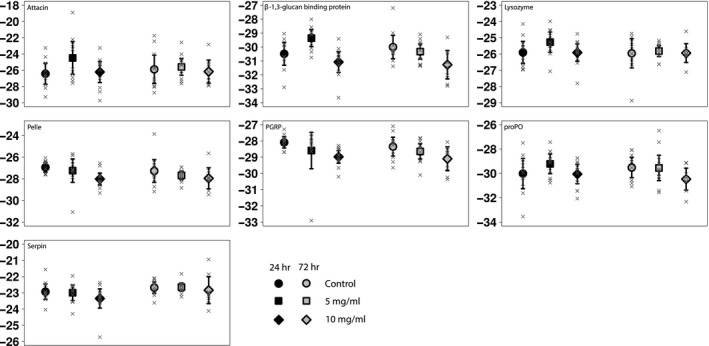
Gene expression upon oral exposure. Average gene expression levels 24 hr (black) and 72 hr (gray) after oral exposure to *M. luteus*. Individuals were either exposed to a control diet (circles), a diet supplemented to 5 mg bacteria per ml (squares), or 10 mg bacteria per ml (diamonds). Error bars indicate 95% confidence intervals around the mean, and separate observations are shown with crosses

Twenty‐four hours postexposure, females exposed to the 5 mg/ml bacterial concentration showed a higher expression of attacin and β‐1,3‐glucan recognition protein by 2.40 ± 1.00 Ct and 1.13 ± 0.51 Ct, respectively, and a 1.04 ± 0.40 Ct higher expression of pelle upon exposure to the 10 mg/ml bacterial exposure, all of which turned nonsignificant after correction for multiple comparisons (Figure 1, Table 1). Seventy‐two hours postexposure to the 10 mg/ml bacterial concentration, females showed a 1.27 ± 0.54 Ct higher expression of β‐1,3‐glucan recognition protein, which also turned nonsignificant after correction for multiple comparisons. Between the two time points, females exposed to the 5 mg/ml bacterial concentration showed a 1.10 ± 0.91 Ct reduced expression of attacin 72 hr postexposure than 24 hr postexposure, which, however, was not statistically significant. None of the other genes showed a difference in gene expression >1.0 Ct between time points, which was also reflected in the absence of statistical significance for these comparisons.

**Table 1:** Gene expression after oral exposure

	*β* ± *SE*	*t*‐value	*p*‐Value	*q*‐Value	*β* ± *SE*	*t*‐Value	*p*‐Value	*q*‐Value
	**Attacin**				**β‐1,3‐glucan recognition protein**			
Control (24 hr) – 5 mg/ml (24 hr)	−2.06 ± 0.94	−2.1928	.0283	0.0991	−1.13 ± 0.47	−2.3845	.0171	0.0991
Control (24 hr) – 10 mg/ml (24 hr)	−0.32 ± 0.89	−0.3528	.7242	0.9382	0.59 ± 0.46	1.284	.1991	0.3485
Control (72 hr) – 5 mg/ml (72 hr)	−0.32 ± 0.89	−0.364	.7159	0.9584	0.32 ± 0.46	0.7012	.4832	0.9584
Control (72 hr) – 10 mg/ml (72 hr)	0.43 ± 0.99	0.4314	.6662	0.7772	1.27 ± 0.50	2.528	.0115	0.0698
Control (24 hr) – Control (72 hr)	−0.64 ± 0.93	−0.6891	.4908	0.6387	−0.48 ± 0.47	−1.0193	.3081	0.6387
5 mg/ml (24 hr) – 5 mg/ml (72 hr)	1.10 ± 0.91	1.2131	.2251	0.3919	0.97 ± 0.46	2.0985	.0359	0.251
10 mg/ml (24 hr) – 10 mg/ml (72 hr)	0.11 ± 0.98	0.1083	.9138	0.9932	0.19 ± 0.49	0.3922	.6949	0.9729
	**Lysozyme**				**Pelle**			
Control (24 hr) – 5 mg/ml (24 hr)	−0.51 ± 0.39	−1.3029	.1926	0.3371	0.28 ± 0.48	0.5816	.5608	0.6543
Control (24 hr) – 10 mg/ml (24 hr)	0.03 ± 0.36	0.0876	.9302	0.9382	1.08 ± 0.45	2.4167	.0157	0.0548
Control (72 hr) – 5 mg/ml (72 hr)	−0.15 ± 0.36	−0.4119	.6804	0.9584	0.48 ± 0.44	1.0757	.2821	0.9584
Control (72 hr) – 10 mg/ml (72 hr)	−0.07 ± 0.41	−0.1618	.8714	0.8714	0.49 ± 0.51	0.9542	.34	0.595
Control (24 hr) – Control (72 hr)	0.10 ± 0.38	0.2681	.7886	0.7886	0.28 ± 0.47	0.6016	.5474	0.6387
5 mg/ml (24 hr) – 5 mg/ml (72 hr)	0.46 ± 0.37	1.2364	.2163	0.3919	0.48 ± 0.46	1.0354	.3005	0.3919
10 mg/ml (24 hr) – 10 mg/ml (72 hr)	0.00 ± 0.40	0.0085	.9932	0.9932	−0.32 ± 0.50	−0.6263	.5311	0.9729
	**PGRP**				**ProPO**			
Control (24 hr) – 5 mg/ml (24 hr)	0.30 ± 0.39	0.7714	.4404	0.6166	−0.80 ± 0.60	−1.3278	.1842	0.3371
Control (24 hr) – 10 mg/ml (24 hr)	0.95 ± 0.33	2.8728	.0041	0.0285	0.05 ± 0.59	0.0776	.9382	0.9382
Control (72 hr) – 5 mg/ml (72 hr)	0.40 ± 0.32	1.2275	.2196	0.9584	0.03 ± 0.59	0.0522	.9584	0.9584
Control (72 hr) – 10 mg/ml (72 hr)	0.92 ± 0.39	2.3274	.0199	0.0698	0.95 ± 0.64	1.4935	.1353	0.3157
Control (24 hr) – Control (72 hr)	0.25 ± 0.35	0.7076	.4792	0.6387	−0.49 ± 0.60	−0.8204	.412	0.6387
5 mg/ml (24 hr) – 5 mg/ml (72 hr)	0.35 ± 0.36	0.9622	.3359	0.3919	0.33 ± 0.59	0.5715	.5677	0.5677
10 mg/ml (24 hr) – 10 mg/ml (72 hr)	0.21 ± 0.39	0.5436	.5867	0.9729	0.41 ± 0.62	0.6621	.5079	0.9729
	**Serpin**							
Control (24 hr) – 5 mg/ml (24 hr)	0.06 ± 0.31	0.2083	.835	0.835				
Control (24 hr) – 10 mg/ml (24 hr)	0.42 ± 0.30	1.3807	.1674	0.3485				
Control (72 hr) – 5 mg/ml (72 hr)	−0.03 ± 0.30	−0.0852	.9321	0.9584				
Control (72 hr) – 10 mg/ml (72 hr)	0.16 ± 0.33	0.4916	.623	0.7772				
Control (24 hr) – Control (72 hr)	−0.25 ± 0.31	−0.8209	.4117	0.6387				
5 mg/ml (24 hr) – 5 mg/ml (72 hr)	−0.35 ± 0.30	−1.1386	.2549	0.3919				
10 mg/ml (24 hr) – 10 mg/ml (72 hr)	−0.51 ± 0.32	−1.5866	.1126	0.7883				

Note

Results from pairwise comparisons of the adjusted Ct values between females that had been exposed to pure honey‐water (control) or honey‐water supplemented with *M. luteus* to a concentration of either 5 mg/ml or 10 mg/ml. Measurements of gene expression were taken 24 and 72 hr postexposure. Estimated differences and standard errors are provided as *β* ± *SE* (negative values indicate lower gene expression in the first treatment) and *q*‐Values indicate the fdr‐adjusted *p*‐values after correction for a comparison of seven genes.

The degree of encapsulation was unaffected by the bacterial exposure treatment (gray value_Control_: 0.037 ± 3.87, gray value_5 mg/ml_: 2.81 ± 3.17, gray value_10 mg/ml_: −2.43 ± 3.22, Table 2).

**Table 2:** Encapsulation response after oral and hemocoelic exposure

	*β* ± *SE*	*df*	*t*‐Value	*p*‐Value
Oral exposure				
Intercept	0.04 ± 3.38	89	0.011	.991
5 mg/ml	2.77 ± 4.82	89	0.574	.567
10 mg/ml	−2.47 ± 4.91	89	−0.503	.616
Hemocoelic exposure				
Intercept	5.55 ± 2.16	22.02	2.567	.0176
PBS	0.73 ± 2.77	78.04	0.262	.7941
5 mg/ml	−15.97 ± 2.75	78.65	−5.815	<.0001

Note

Results from linear mixed‐effects regression on gray values (which reflect the degree of melanization of the inserted monofilament). Differences in gray values to the reference level (Intercept = Control) are given as *β* ± *SE*, including standard errors.

#### 3.1.2 Life history traits

**Figure 2:**
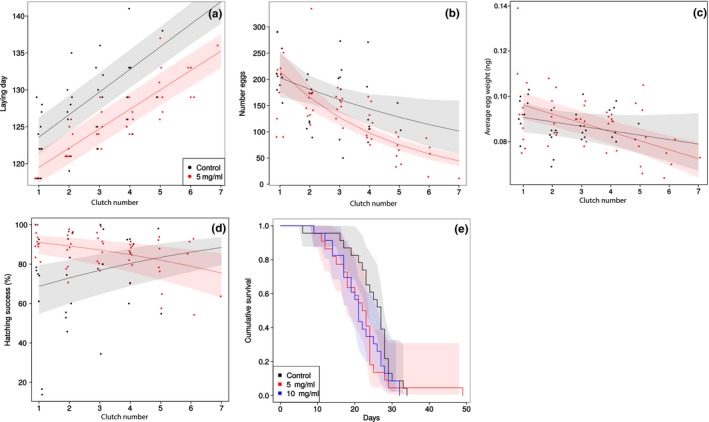
life history changes upon oral exposure. Separate panels show (a) the time of egg laying of each clutch reflected as day of the year, (b) the amount of eggs per clutch, (c) the average weight per egg for each clutch, (d) the hatching success in percentage of each clutch, and (e) the cumulative survival of females. For panels a–d, black dots indicate clutches laid by females exposed to a control diet and red dot clutches from females exposed to a diet supplemented to a final concentration of 5 mg bacteria per ml. The points are jittered on the *x*‐axis to improve visibility, but naturally can only take discrete values. The lines reflect predicted egg laying times for each group (95% confidence intervals are shaded), based on a mixed‐effects linear regression model. Panel E additionally features data for females exposed to a diet supplemented to a final concentration of 10 mg bacteria per ml (in blue)

Females that had been exposed to bacteria (5 mg/ml) induced egg laying significantly earlier than control females, with an average difference of 4.09 ± 1.61 days (Figure 2a, Table 3). The average amount of days between clutches was 3.06 ± 0.18 for control females and 2.63 ± 0.11 for bacteria‐exposed females, which constituted a significant difference in the egg laying interval between treatments.

**Table 3:** life history traits after oral exposure

Laying time, lmer	*β* ± *SE*	*df*	*t*‐value	*p*‐Value
Intercept	123.57 ± 1.18	18.8	105.107	<.0001
Treatment	−4.09 ± 1.61	18.5	−2.532	.0206
Clutch number	3.06 ± 0.18	62.4	16.749	<.0001
Treatment × Clutch number	−0.43 ± 0.21	62.2	−2.056	.0439

Number clutches, vglm	*β* ± *SE*		*z*‐value	*p*‐Value
Intercept 1	−1.66 ± 0.96			
Intercept 2	1.77 ± 0.2		8.672	<.0001
Treatment	0.36 ± 0.13		2.779	.00545

Number eggs, glmer.nb	*β* ± *SE*		*z*‐value	*p*‐Value
Intercept	5.32 ± 0.1		53.222	<.0001
Treatment	0.06 ± 0.13		0.416	.6777
Clutch number	−0.12 ± 0.05		−2.277	.0228
Treatment × Clutch number	−0.15 ± 0.06		−2.395	.0166

Lifetime egg production, Wilcoxon rank sum test	*β* ± *SE*		*W*	*p*‐Value
Treatment (Control)	614.0 ± 83.9			
Treatment (5 mg/ml)	655.3 ± 35.66		40	.72

Egg weight, lmer	*β* ± *SE*	*df*	*t*‐value	*p*‐Value
Intercept	0.09 ± 0.003	33	28.13	<.0001
Treatment	0.005 ± 0.004	31.4	1.249	.221
Clutch number	−0.002 ± 0.001	68.9	−1.596	.115
Treatment × Clutch number	−0.002 ± 0.001	68.1	−1.378	.173

Hatching success, glmer (binom)	*β* ± *SE*		*z*‐value	*p*‐Value
Intercept	0.79 ± 0.28		2.803	.00506
Treatment	1.53 ± 0.39		3.927	<.0001
Clutch number	0.21 ± 0.03		6.283	<.0001
Treatment × Clutch number	−0.41 ± 0.04		−9.469	<.0001

Longevity, coxme	*β* ± *SE*	HR	*z*‐value	*p*‐Value
Treatment (5 mg/ml)	0.45 ± 0.33	1.57	1.38	.17
Treatment (10 mg/ml)	0.62 ± 0.32	1.86	1.97	.049

Note

Results from the statistical analyses on various life‐history traits after oral exposure. For each life‐history trait, the type of model is indicated (for details, see the Statistical analysis section). Parameter estimates and standard errors are provided as *β* ± *SE*. Interaction terms are indicated with an × between parameters. For all models, Treatment was a factor (control, 5 mg/ml) and Clutch number a continuous covariate.

Bacteria‐exposed females produced on average more clutches than control females (control: 3.44 ± 0.44 clutches, bacteria: 5.00 ± 0.45 clutches; Table 3).

However, the amount of eggs did not differ between treatments for first clutches (control: 220.56 ± 16.15 eggs, bacteria: 179.90 ± 18.28 eggs; Figure 2b), but decreased significantly faster with increasing clutch rank for bacteria‐exposed females (23% decline per clutch) than control females (11% decline per clutch). Lifetime egg production did not show a strong difference between control females and bacteria‐exposed females (control: 614.0 ± 83.9 eggs, bacteria: 655.3 ± 35.66 eggs; Table 3).

The average weight of first‐clutch eggs did not differ between treatments (control: 0.094 ± 0.003 ng, bacteria: 0.095 ± 0.006 ng; Figure 2c, Table 3), and the decrease in egg weight with increasing clutch rank was negligible (control: −0.002 ± 0.001 ng per clutch, bacteria: −0.004 ± 0.001 ng per clutch). Hatching success of first clutches was initially higher in clutches of bacteria‐exposed females (control: 65.37 ± 10.19% hatched, bacteria: 90.38 ± 1.74% hatched; Figure 2d, Table 3). However, for clutches of control females, hatching success increased with increasing clutch rank by 3.73 ± 0.59% per clutch, whereas hatching success of clutches from bacteria‐exposed females decreased by 2.19 ± 0.30% per clutch.

The 5 mg/ml concentration did not have a significant influence on female life span compared to the control exposure (HR = 1.57; Figure 2e, Table 3) but the 10 mg/ml concentration increased the risk of mortality compared to the control (HR = 1.86), yet the associated p‐value was only marginally lower than our alpha level (*p* = .049).

### 3.2 Hemocoelic bacterial exposure

#### 3.2.1 Immune defense

**Figure 3:**
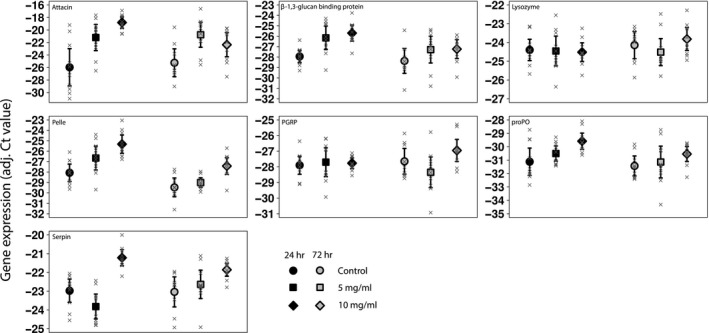
Gene expression upon hemocoelic exposure. Average gene expression levels 24 hr (black) and 72 hr (gray) after hemocoelic exposure. Individuals were either only pierced as a control (circles), injected with pure PBS (squares), or injected with 5 mg/ml bacteria PBS solution (diamonds). Error bars indicate 95% confidence intervals around the mean, and separate observations are shown with crosses

Twenty‐four hours postinjection of bacteria, females showed an up‐regulation of the genes attacin (7.18 ± 1.28 Ct), β‐1,3‐glucan recognition protein (2.24 ± 0.26 Ct), pelle (2.75 ± 0.53 Ct), proPO (1.77 ± 0.46 Ct), and serpin (1.76 ± 0.38 Ct; Table 4, Figure 3), all of which were statistically significant. Four of these genes, attacin, β‐1,3‐glucan recognition protein, pelle, and serpin, were also up‐regulated 24 hr postinjection of PBS, however, to a smaller degree (attacin: 4.89 ± 1.31 Ct; β‐1,3‐glucan recognition protein: 1.79 ± 0.62 Ct; pelle: 1.42 ± 0.53 Ct; serpin: 0.84 ± 0.38 Ct).

**Table 4:** Gene expression after hemocoelic exposure

	*β* ± *SE*	*t*‐Value	*p*‐Value	*q*‐Value	*β* ± *SE*	*t*‐Value	*p*‐Value	*q*‐Value
	**Attacin**				**β‐1,3‐glucan recognition protein**			
Control (24 hr) – PBS (24 hr)	−4.89 ± 1.31	−3.73	.0002	0.0013	−1.79 ± 0.62	−2.89	.0039	0.0136
Control (24 hr) – 5 mg/ml (24 hr)	−7.18 ± 1.28	−5.59	0	0	−2.24 ± 0.62	−3.63	.0003	0.0004
Control (72 hr) – PBS (72 hr)	−4.41 ± 1.33	−3.32	.0009	0.0063	−1.09 ± 0.64	−1.71	.0874	0.2563
Control (72 hr) – 5 mg/ml (72 hr)	−3.07 ± 1.32	−2.33	.0199	0.0464	−1.14 ± 0.64	−1.79	.0734	0.1027
Control (24 hr) – Control (72 hr)	−0.81 ± 1.32	−0.62	.5377	0.796	0.43 ± 0.64	0.68	.4991	0.796
PBS (24 hr) – PBS (72 hr)	−0.32 ± 1.30	−0.25	.8036	0.8036	1.13 ± 0.62	1.83	.068	0.1586
5 mg/ml (24 hr) – 5 mg/ml (72 hr)	3.30 ± 1.29	2.56	.0105	0.0306	1.53 ± 0.62	2.48	.0131	0.0306
	**Lysozyme**				**Pelle**			
Control (24 hr) – PBS (24 hr)	0.04 ± 0.39	0.11	.9145	0.9145	−1.42 ± 0.53	−2.67	.0076	0.0177
Control (24 hr) – 5 mg/ml (24 hr)	0.23 ± 0.37	0.63	.5294	0.6176	−2.75 ± 0.53	−5.18	0	0
Control (72 hr) – PBS (72 hr)	0.51 ± 0.38	1.33	.1831	0.2563	−0.46 ± 0.54	−0.85	.3956	0.3956
Control (72 hr) – 5 mg/ml (72 hr)	−0.12 ± 0.38	−0.31	.7533	0.7533	−2.05 ± 0.54	−3.76	.0002	0.0012
Control (24 hr) – Control (72 hr)	−0.15 ± 0.38	−0.41	.6823	0.796	1.40 ± 0.54	2.57	.0102	0.0713
PBS (24 hr) – PBS (72 hr)	0.32 ± 0.39	0.80	.4214	0.5899	2.35 ± 0.53	4.44	0	0.0001
5 mg/ml (24 hr) – 5 mg/ml (72 hr)	−0.51 ± 0.37	−1.36	.175	0.175	2.10 ± 0.53	3.96	.0001	0.0005
	**PGRP**				**ProPO**			
Control (24 hr) – PBS (24 hr)	−0.21 ± 0.47	−0.44	.659	0.7689	−0.63 ± 0.49	−1.29	.1972	0.276
Control (24 hr) – 5 mg/ml (24 hr)	−0.14 ± 0.47	−0.29	.7683	0.7683	−1.77 ± 0.46	−3.83	.0001	0.0002
Control (72 hr) – PBS (72 hr)	0.68 ± 0.48	1.42	.1563	0.2563	−0.75 ± 0.48	−1.57	.1175	0.2563
Control (72 hr) – 5 mg/ml (72 hr)	−0.68 ± 0.48	−1.43	.1538	0.1795	−0.98 ± 0.47	−2.10	.0356	0.0622
Control (24 hr) – Control (72 hr)	−0.24 ± 0.48	−0.49	.6213	0.796	0.32 ± 0.47	0.67	.5015	0.796
PBS (24 hr) – PBS (72 hr)	0.65 ± 0.47	1.39	.1644	0.2877	0.20 ± 0.49	0.40	.6887	0.8035
5 mg/ml (24 hr) – 5 mg/ml (72 hr)	−0.78 ± 0.47	−1.68	.0939	0.1096	1.10 ± 0.46	2.37	.0176	0.0307
	**Serpin**							
Control (24 hr) – PBS (24 hr)	0.84 ± 0.38	2.20	.0275	0.0482				
Control (24 hr) – 5 mg/ml (24 hr)	−1.76 ± 0.38	−4.61	0	0				
Control (72 hr) – PBS (72 hr)	−0.40 ± 0.39	−1.02	.3066	0.3577				
Control (72 hr) – 5 mg/ml (72 hr)	−1.18 ± 0.39	−3.01	.0026	0.0092				
Control (24 hr) – Control (72 hr)	0.06 ± 0.39	0.15	.8786	0.8786				
PBS (24 hr) – PBS (72 hr)	−1.18 ± 0.38	−3.10	.002	0.0068				
5 mg/ml (24 hr) – 5 mg/ml (72 hr)	0.64 ± 0.38	1.68	.0934	0.1096				

Note

Results from pairwise comparisons of the adjusted Ct values between females that had been sham treated (Control) injected with PBS or injected with 5 mg/ml *M. luteus*. Measurements of gene expression were taken 24 and 72 hr postexposure. Estimated differences and standard errors are provided as *β* ± *SE* (negative values indicate lower gene expression in the first treatment) and *q*‐values indicate the fdr‐adjusted *p*‐values after correction for a comparison of seven genes.

The genes attacin, pelle, and serpin were also up‐regulated 72 hr postbacterial injection of bacteria into females (attacin: 3.07 ± 1.32 Ct; pelle: 2.05 ± 0.54 Ct; serpin: 1.18 ± 0.39 Ct). Additionally, females showed an up‐regulation of attacin by 4.41 ± 1.33 Ct upon injection of PBS at this time point.

Control females showed a lower expression of pelle by 1.40 ± 0.54 Ct 72 hr compared to 24 hr post‐treatment, which turned nonsignificant after correction for multiple comparisons. Bacteria‐injected females showed lower expression of attacin, β‐1,3‐glucan recognition protein, pelle, and proPO by 3.30 ± 1.29 Ct, 1.53 ± 0.62 Ct, 2.10 ± 0.53 Ct, and 1.10 ± 0.46 Ct, respectively. When injected with PBS, females showed reduced expression of pelle by 2.35 ± 0.53 Ct. In contrast, serpin was 1.18 ± 0.38 Ct more expressed 72h compared to 24h postinjection in females.

Injection of bacteria resulted in a significantly stronger encapsulation response than the control treatment or the PBS treatment (gray value_Control_: 5.16 ± 1.69, gray value_PBS_: 6.24 ± 1.94, gray value_5 mg/ml_: −10.55 ± 2.25; Table 2).

#### 3.2.2 Life history traits

**Figure 4:**
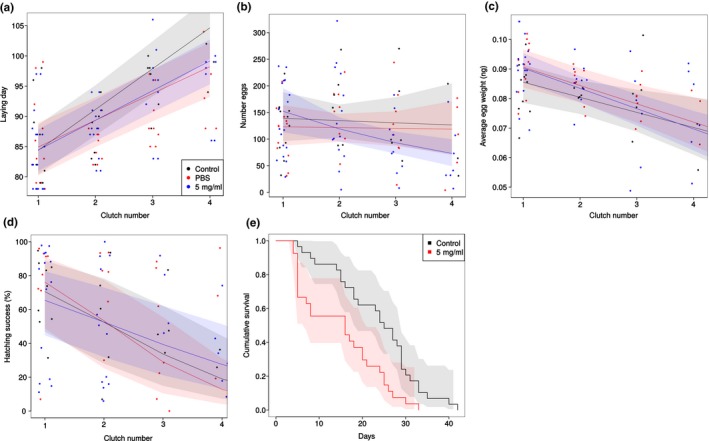
Timing of egg laying life‐history changes upon oral exposure. Separate panels show (a) the time of egg laying of each clutch reflected as day of the year, (b) the amount of eggs per clutch, (c) the average weight per egg for each clutch, (d) the hatching success in percentage of each clutch, and (e) the cumulative survival of females. For panels a–d, black dots indicate clutches laid by control females (only pierced but not injected), blue dots indicate clutches from females injected with a pure PBS solution, and red dots correspond to clutches from females injected with a 5 mg bacteria per ml PBS solution. The points are jittered on the *x*‐axis to improve visibility, but naturally can only take discrete values. The lines reflect predicted egg laying times for each group (95% confidence intervals are shaded), based on a mixed‐effects linear regression model. Panel e does not feature the PBS control, as this was omitted for this experiment

The timing of egg laying of first clutches did not differ between treatments (control–PPS: 0.05 ± 2.72 days of difference, control–bacteria 0.55 ± 2.94 days of difference; Figure 4a, Table 5). However, females that had been injected with PBS or bacteria showed shorter intervals between clutches than control females (control: 6.76 ± 0.56 days, PBS: 4.91 ± 0.41 days, bacteria: 4.43 ± 0.47 days).

**Table 5:** Life history traits after hemocoelic exposure

Laying time, lmer	*β* ± *SE*	*df*	*t*	*p*
Intercept	84.38 ± 2.13	37.7882	39.622	<.0001
Treatment (PBS)	0.28 ± 2.72	37.2416	0.102	.91946
Treatment (5 mg/ml)	0.55 ± 2.95	37.6748	0.187	.85291
Clutch number	6.76 ± 0.56	69.0359	11.981	<.0001
Treatment (PBS) × Clutch number	−2.19 ± 0.65	68.8617	−3.373	.00122
Treatment (5 mg/ml) × Clutch number	−2.33 ± 0.74	68.7567	−3.165	.00231

Number clutches, glm	*β* ± *SE*	*df*	*z*	*p*
Intercept	0.90 ± 0.19		4.666	<.0001
Treatment (PBS)	0.20 ± 0.24		0.843	.399
Treatment (5 mg/ml)	0.11 ± 0.26		0.438	.661

Number eggs, glmer.nb	*β* ± *SE*	*df*	*z*	*p*
Intercept	4.94 ± 0.18		28.067	<.0001
Treatment (PBS)	0.09 ± 0.22		0.406	.685
Treatment (5 mg/ml)	−0.12 ± 0.24		−0.506	.613
Clutch number	−0.03 ± 0.12		−0.257	.797
Treatment (PBS) × Clutch number	−0.21 ± 0.15		−1.394	.163
Treatment (5 mg/ml) × Clutch number	0.02 ± 0.16		0.12	.905

Lifetime egg production, ANOVA	*β* ± *SE*	*df*	*F*	*p*‐Value
Treatment (Control)	630.64 ± 73.71			
Treatment (PBS)	352.41 ± 74.66			
Treatment (5 mg/ml)	333.75 ± 66.64	2	0.041	.96

Weight, lmer	*β* ± *SE*	*df*	*t*	*p*
Intercept	0.085 ± 0.003	54.751426	26.103	<.0001
Treatment (PBS)	0.003 ± 0.004	50.7013309	0.819	.4166
Treatment (5 mg/ml)	0.005 ± 0.005	51.5644177	1.151	.25518
Clutch number	−0.005 ± 0.002	64.8503645	−2.674	.00947
Treatment (PBS) × Clutch number	0.001 ± 0.002	64.9127958	0.297	.76755
Treatment (5 mg/ml) × Clutch number	−0.001 ± 0.002	62.5879687	−0.481	.632

Hatching success, glmer (binom)	*β* ± *SE*	*df*	*z*	*p*
Intercept	0.87 ± 0.53		1.647	.09954
Treatment (PBS)	−0.30 ± 0.68		−0.445	.65654
Treatment (5 mg/ml)	0.29 ± 0.78		0.373	.70897
Clutch number	−0.78 ± 0.06		−12.77	<.0001
Treatment (PBS) × Clutch number	0.38 ± 0.07		5.2	<.0001
Treatment (5 mg/ml) × Clutch number	−0.25 ± 0.08		−3.112	.00186

Longevity, coxme	*β* ± *SE*	exp(coef)	*z*	*p*
Treatment (5 mg/ml)	0.89 ± 2.44	2.437209	2.88	.004

Note

Results from the statistical analyses on various life‐history traits after hemocoelic exposure. For each life‐history trait, the type of model is indicated (for details, see the Statistical analysis section). Parameter estimates and standard errors are provided as *β* ± *SE*. Interaction terms are indicated with an × between parameters. For all models, Treatment was a factor (control, PBS, 5 mg/ml) and Clutch number a continuous covariate.

Treatment did not significantly affect the amount of clutches produced by females (control: 2.46 ± 0.39 clutches, PBS: 3.00 ± 0.38 clutches, bacteria: 2.75 ± 0.39 clutches; Table 5). Although the number of eggs decreased in all treatments with clutch rank (control: −3% per clutch, PBS: −25% per clutch, bacteria: −1% per clutch; Figure 4b), the decrease was revealed not to be statistically significant. Consequentially, lifetime egg production did not differ significantly among the treatments (control: 330.64 ± 73.71, PBS: 352.41 ± 47.66, bacteria: 333.75 ± 66.64; Table 5).

The weight of the eggs decreased with clutch rank in all treatments (control: −0.005 ± 0.002 ng per clutch, PBS: −0.007 ± 0.001 ng per clutch, bacteria: −0.006 ± 0.002 ng per clutch; Figure 4c, Table 5). Hatching success of first clutches did not differ significantly among treatments (control: 69.09 ± 6.93% hatched, PBS: 63.43 ± 9.38% hatched, bacteria: 75.33 ± 10.34% hatched; Figure 4d, Table 5). Hatching success decreased significantly with increasing clutch rank, and injection of bacteria resulted in a faster decline of hatching success over time, whereas injection of PBS had the opposite effect (control: −19.4 ± 1.52% per clutch, PBS: −13.4 ± 1.28% per clutch, bacteria: −25.7 ± 1.33% per clutch; Table 5).

Female life span was significantly reduced by injection of bacteria as compared to the naive control (females HR = 2.44; Figure 4e, Table 5).

## 4 DISCUSSION

Here, we studied the impact of two different modes of infection, oral exposure and hemocoelic exposure, on life histories and immune defenses of the butterfly *M. cinxia*. Infection in general resulted in shorter intervals between clutches and hatching success decreased when the females had been exposed to bacteria, as did the life span of the females. However, the strength of these responses differed between the two modes of infection. Furthermore, certain life histories were only detectable for one type of infection. Reductions in the size of clutches and earlier production of first clutches also emerged only when the females had been orally exposed, whereas hemocoelic exposure resulted in a decrease of average egg weight with increasing clutch number. In terms of immunity, only hemocoelic exposure resulted in statistically detectable changes in gene expression and encapsulation response; however, the genes with the strongest change were the same for both infection modes, namely attacin, β‐1,3‐glucan recognition protein, and pelle.

For most genes, infected females showed higher gene expression of the selected immunity‐related genes than uninfected females, which was less pronounced for oral exposure than for hemocoelic exposure. This is not unexpected, as the gut provides an additional chemical and physical barrier against infections through enzymes and the gut epithelium (Ferrandon et al., 2007; Vallet‐Gely et al., 2008). Hence, the hemocoelic infection presents a more imminent danger and should provoke a stronger response. Alternatively, and not mutually exclusive, the injury accompanying the hemocoelic infection may have added additional stress and resulted in an interaction with the immune response (Theopold et al., 2002). For both exposure types, the strongest response was found for the same genes, attacin, β‐1,3‐glucan recognition protein, and pelle, although the changes after oral exposure were not statistically significant. As the changes were more than twofold (>1 Ct) for each of them and their function match previous findings in other study systems, we nevertheless consider them to be at least weakly induced, despite the absence of statistical support. A response in β‐1,3‐glucan recognition protein and pelle can be expected, as both genes are part of the defense pathway against Gram‐positive bacteria (Brown & Gordon, 2005; Medzhitov & Janeway, 2000). The antimicrobial peptide attacin is predominantly involved in the defense against Gram‐negative bacteria (Carlsson et al., 1998). Nevertheless, attacin has been shown to also be up‐regulated in *D. melanogaster* upon exposure to *M. luteus* and the fungus *Beauveria bassiana* (Lemaitre, Reichhart, & Hoffmann, 1997). This may indicate a cross‐talk between the different pathways (Tanji, Hu, Weber, & Ip, 2007).

The effect of infection on reproductive success varies across insect taxa (Adamo, 1999; Ahmed et al., 2002; Calleri, Rosengaus, & Traniello, 2006; Sylvestre, Gandini, & Maciel‐de‐freitas, 2013), however, usually is considered to be subject to a trade‐off between immune defenses and reproduction (Flat & Heyland, 2011; Schmid‐Hempel, 2011). Here, this trade‐off was reflected in reduced hatching success of offspring from infected females. Decreasing hatching success is generally considered an effect of aging, and infections often accelerate aging (Pursall & Rolff, 2011). Such accelerated senescence may also be reflected in the shortened life span of infected females; however, it is impossible here to distinguish between mortality due to accelerated senescence and mortality caused by other stress from the infections (e.g., opportunistic infections). The decline in hatching success may correlate with the observation that orally exposed females induced egg laying earlier and females of both treatments showed shorter intervals between clutches. Infections often lead to an earlier onset of reproduction, due to the increased extrinsic mortality risk (Adamo, 1999). As a result, infected females may not be able to immediately invest as many resources into egg production, which may reduce offspring quality, and hence, hatching success. As the average weight of the eggs did not appear to be affected by the infection, this decrease in hatching success may have originated from a reduction in the quality of the provided nutrients or other yolk components (Geister, Lorenz, Hoffmann, & Fischer, 2008). Further studies on the precise relationship between infection and egg quality are required, to unveil the mechanisms driving hatching success as a life‐history trait. Interestingly, lifetime egg production was similar across treatments, which may suggest that any trade‐off is temporally confined. Thus, the females may be constrained in their resource investment at any one time, but may ultimately be able to compensate by producing more clutches.

Despite the stronger immune response upon hemocoelic exposure, the alterations in many life‐history traits appeared less pronounced in hemocoelically exposed than orally exposed females. This may be a simple family effect, as the involved individuals did not originate from the same families. Alternatively, the result may indicate fecundity compensation by the infected individuals. Fecundity compensation allows individuals who suffer from a reduced residual life span to compensate for their expected loss in available time for reproduction. Compensation is usually achieved by starting reproduction earlier (Jokela & Lively, 1995; Leventhal, Dünner, & Barribeau, 2014) or increasing the reproductive output per unit of time (Adamo, 1999; Krist, 2001). Our results reflect similar patterns. In both, oral and hemocoelic exposure, infected individuals showed shorter intervals between clutches, and orally exposed individuals started reproduction earlier than their respective control. Nevertheless, hemocoelic exposure resulted in a slightly higher hazard rate than oral exposure, which reduced the total amount of clutches compared to orally exposed butterflies. Because hemocoelic exposure in contrast had a smaller effect on clutch size and the hatching success, this may reflect different strategies for fecundity compensation (Leventhal et al., 2014). In their study, Leventhal et al. (2014) report that delayed virulence and low costs to an infection may promote fecundity compensation. This concurs with our observation that the less harmful mode of infection, oral exposure, resulted in slightly stronger alterations to life histories. It may be interesting for future studies to investigate the effect of dose on the alteration of life histories. Such an experiment could shed more light on the precise relationship between mode of infection, the strength of the immune defense, and the adaptation to life‐history strategies. Based on the results of this study, we would predict higher doses for oral exposure to lead to similar results as the hemocoelic exposure in this study, and vice versa.

Another potential mechanism factoring into the observed effects may be infection‐induced anorexia (Ayres & Schneider, 2009). In particular, when the bacteria were fed to the females, the individuals may have limited their exposure by reducing their food consumption (yet individuals may also lose appetite when infected by other means). Given that this may ultimately have reduced their overall nutrient uptake, alterations in fecundity and other life‐history traits may also arise through indirect effects of exposure to bacteria. Therefore, precise measurement of food consumption (and ideally bacterial load) is necessary to reveal the underlying mechanisms to the observed effects. Furthermore, we cannot exclude influences from nonrandom mating. Given that the mating rate was much lower in the hemocoelic exposure experiment, compared to the oral exposure experiment, it is possible that differences between the experiments may have been affected by the initial quality of the individuals.

Changes in the environmental conditions can lead to changes in host–parasite interactions (Hatcher et al., 2006). For example, warmer environmental conditions lead to higher metabolic rates for both host and parasite (Kirk et al., 2018). Should this effect be asynchronous, one or the other party may gain advantage in the arms race, leading to permanent adaptations in life histories of the host. Similarly, changing environmental conditions alter the behavioral activity of the host, such as the time spent flying (Douwes, 1976), or the palatability to predators (Srygley & Chai, 1990). As these behaviors can influence the risk of wounding and the exposure to contaminated soil and food, this may lead to changes in the prior mode of how parasites are encountered and contracted. Given the here demonstrated differences between oral and hemocoelic exposure on the life histories of *M. cinxia*, shifts in host–parasite interactions due to environmental change may alter the predominant expression of life histories within different populations. Depending on the prevalence of parasites, strong selection pressure may contribute to the development of synchrony (Kahilainen, Nouhuys, Schulz, & Saastamoinen, 2018) in population dynamics. Interactions between mode of transmission, environmental conditions, and life histories have already been shown for horizontal and vertical modes of transmission/infection (Agnew & Koella, 1999; Vizoso & Ebert, 2005). By contrast, the relationship between different horizontal modes of transmission and life‐history alterations has only received recent attention. A recent study on *D. melanogaster* found no indication of life‐history alterations (total reproductive output, starvation and desiccation resistance, development time) after 30 generations' exposure to different infection routes (Faria et al., 2015). However, this was to our knowledge the only study so far that directly tested whether different horizontal infection routes have a long‐term influence on life histories. We therefore advocate more research to be conducted, in order to properly investigate how different horizontal routes of infection may affect life‐history evolution.

In conclusion, our results indicate that infections can be a short‐term driver of life histories and that different modes of infection can result in different expressions of life‐history traits. The most likely explanation for the differences between the infection modes lies in the severity of the emerging infection. A direct injection likely ends in a systemic infection, whereas an infection through the gut may be localized and fended off more easily. Hence, the alterations to life‐history may be linked to the residual life span and therefore reflect different strategies for fecundity compensation. As a result, alterations in the prevalence of parasites and their mode of infection may have a strong impact on a host's life histories and ultimately shape the population dynamics of the host.

## CONFLICT OF INTEREST

The authors declare no conflicts of interests.

## AUTHOR CONTRIBUTIONS

LW and MS designed the study; LW performed the experiments; DS and LW analyzed the data; DS, LW, and MS wrote the paper. All authors contributed critically to the drafts and gave final approval for publication.

## Data Availability

The datasets supporting this article and the required R script to replicate the analyses are deposited on Dryad (https://doi.org/10.5061/dryad.ft3qd24).
